# SS-DRPL: self-supervised deep representation pattern learning for voice-based Parkinson's disease detection

**DOI:** 10.3389/fncom.2024.1414462

**Published:** 2024-06-12

**Authors:** Tae Hoon Kim, Moez Krichen, Stephen Ojo, Gabriel Avelino Sampedro, Meznah A. Alamro

**Affiliations:** ^1^School of Information and Electronic Engineering, Zhejiang University of Science and Technology, Hangzhou, Zhejiang, China; ^2^FCSIT, Al-Baha University, Al-Baha, Saudi Arabia; ^3^Department of Electrical and Computer Engineering, College of Engineering, Anderson University, Anderson, SC, United States; ^4^Faculty of Information and Communication Studies, University of the Philippines Open University, Los Baños, Philippines; ^5^Gokongwei College of Engineering, De La Salle University, Manila, Philippines; ^6^Department of Information Technology, College of Computer and Information Science, Princess Nourah Bint Abdul Rahman University, Riyadh, Saudi Arabia

**Keywords:** Parkinson's disease, artificial intelligence, self-supervised deep representation pattern learning, machine learning, FT-HV

## Abstract

Parkinson's disease (PD) is a globally significant health challenge, necessitating accurate and timely diagnostic methods to facilitate effective treatment and intervention. In recent years, self-supervised deep representation pattern learning (SS-DRPL) has emerged as a promising approach for extracting valuable representations from data, offering the potential to enhance the efficiency of voice-based PD detection. This research study focuses on investigating the utilization of SS-DRPL in conjunction with deep learning algorithms for voice-based PD classification. This study encompasses a comprehensive evaluation aimed at assessing the accuracy of various predictive models, particularly deep learning methods when combined with SS-DRPL. Two deep learning architectures, namely hybrid Long Short-Term Memory and Recurrent Neural Networks (LSTM-RNN) and Deep Neural Networks (DNN), are employed and compared in terms of their ability to detect voice-based PD cases accurately. Additionally, several traditional machine learning models are also included to establish a baseline for comparison. The findings of the study reveal that the incorporation of SS-DRPL leads to improved model performance across all experimental setups. Notably, the LSTM-RNN architecture augmented with SS-DRPL achieves the highest F1-score of 0.94, indicating its superior ability to detect PD cases using voice-based data effectively. This outcome underscores the efficacy of SS-DRPL in enabling deep learning models to learn intricate patterns and correlations within the data, thereby facilitating more accurate PD classification.

## 1 Introduction

The neurological disorder known as Parkinson's disease (PD) has a substantial impact on a considerable proportion of the worldwide population (Muangpaisan et al., 2011; Rocca, 2018). The dopaminergic neurons in the substantia nigra region of the brain gradually deteriorate, which distinguishes them. As a consequence, individuals have motor symptoms, including tremors, bradykinesia, stiffness, and postural instability, which have a substantial influence on their daily functioning and overall quality of life. Due to the chronic nature of the condition and the way these symptoms are advancing over time, comprehensive therapy and care are necessary. Timely detection and prompt intervention play a critical role in minimizing the consequences of PD and maximizing the effectiveness of treatment. The ability to identify the subtle indications and manifestations of PD at its early stages enables the implementation of suitable therapeutic measures with the goal of mitigating symptoms, maintaining motor abilities, and enhancing the overall quality of life (Kanagaraj et al., 2024; Xia et al., 2024).

Individuals with PD may benefit from early intervention measures such as lifestyle adjustments, rehabilitative therapy, and pharmaceutical interventions that are specifically designed to address their unique requirements and limitations (Olanrewaju et al., 2014; Bhat et al., 2018; Lang and Espay, 2018). In order to effectively address a broader spectrum of non-motor symptoms, including cognitive impairment, psychiatric symptoms, and autonomic dysfunction, neurologists, movement disorder specialists, rehabilitation therapists, nurses, social workers, and other healthcare professionals must collaborate in a multidisciplinary approach to care (Seppi et al., 2019). The progress made in diagnostic technology, neuroimaging techniques, and biomarker identification has greatly contributed to the timely and precise identification of PD, allowing healthcare practitioners to intervene during the initial phases of the condition. Nevertheless, there are still obstacles that remain, such as the requirement for more accurate diagnostic biomarkers, the advancement of neuroprotective medicines, and the enhancement of customized therapy approaches for each patient. The increasing societal and economic impact of PD highlights the imperative for ongoing research, lobbying, and public awareness campaigns aimed at improving healthcare accessibility, enhancing treatment efficacy, and finally achieving a cure for this incapacitating disorder (Liu et al., 2024; Sardar and Pahari, 2024).

Recent developments in the field of deep learning have demonstrated potential for enhancing the precision and effectiveness of PD identification (Ali et al., 2023). The use of Self Supervised Representation learning (SSRL) has become increasingly prominent as an effective methodology for acquiring representations from data that lacks labels (Ericsson et al., 2021). The SSRL technique has become a reliable approach for extracting significant representations from data that lacks labels. This method shows potential for enhancing the effectiveness of voice-based PD detection systems. SSRL fundamentally entails acquiring representations from data without the need for explicit supervision (Mohamed et al., 2022). SSRL algorithms differ from standard supervised learning approaches in that they can predict a specific part of the input data based on another part without requiring explicitly defined target outputs. The utilization of this self-supervised methodology allows models to exploit the intrinsic structure and patterns that exist within the data, resulting in enhanced learning efficiency and effectiveness. The success of SSRL algorithms in several areas is indicative of their adaptability since they have exhibited the capacity to improve the efficiency of learning tasks by enhancing sample efficiency (Zhang et al., 2017).

SSRL shows potential in the field of voice-based PD identification. Through the utilization of unlabeled data, these algorithms can reveal latent patterns and characteristics that are suggestive of PD, consequently enhancing the precision of predictive models (Nekoui and Cheng, 2021). It is noteworthy that the numerous benefits of improved PD identification are made possible by self-supervised deep representational learning (Jiang et al., 2021). The timely identification and Early diagnosis of PD is very important so that treatments can be started right away, which could slow the disease's progression and improve patient outcomes (Mei et al., 2021; Öksüz et al., 2022). SSRL algorithms are very important for making detection methods more accurate and reliable, which speeds up the process of early diagnosis and allows healthcare practitioners to intervene during the initial phases of the disease. Moreover, the economic and systemic consequences of improved Parkinson's disease identification are of utmost importance. The utilization of more precise diagnostic instruments not only leads to enhanced patient care but also holds the capacity to substantially mitigate healthcare expenses linked to misdiagnosis or delayed diagnosis (Painuli et al., 2022). SSRL-driven breakthroughs in PD detection enhance healthcare delivery by simplifying the diagnostic procedure and increasing the efficiency of healthcare systems. Furthermore, the utilization of SSRL approaches not only enhances the precision of PD identification but also exhibits the potential for furthering our comprehension of the fundamental mechanisms associated with this condition. These algorithms offer valuable insights into the subtle connections and biomarkers linked to voice-based PD by revealing intricate patterns and representations within unlabeled data. This enhanced comprehension can contribute to the advancement of therapeutic approaches and therapies designed to alleviate the consequences of the condition (Güvenç Paltun et al., 2021).

**Motivation:** PD is a significant healthcare challenge, demanding precise and timely diagnostic methods for effective intervention. Traditional approaches may lack accuracy, prompting the exploration of advanced techniques like self-supervised deep representation pattern learning (SS-DRPL) to extract meaningful features from voice data. By integrating SS-DRPL with deep learning algorithms, this study aims to enhance the accuracy of voice-based PD detection. Evaluation of various machine learning models seeks to identify optimal approaches for early PD identification, ultimately improving patient outcomes through timely intervention and personalized treatment strategies.

**Contributions:** This paper makes the following contributions.

- **Self-supervised deep representational pattern learning technique:** Propose Self-supervised representational pattern learning techniques for the extraction of latent features and structures from data, resulting in enhanced discriminative capabilities of the models. Self-supervised learning has enabled the more efficient utilization of available data in prediction for Parkinson's disease detection by predicting one part of the input data based on another part without relying on manually provided target outputs.
- **Voice-based PD classification with machine learning classifier:** Proposed SS-DRPL based LSTM-RNN, DNN, trained on voice dataset for the detection of PD. The DNN demonstrated a performance of 0.85% in terms of accuracy. The LSTM-RNN, with a score of 0.93%, addresses the issue of overfitting and enhances generalization by integrating many models. Likewise, it resulted in improved predicted accuracy. The LSTM-RNN model demonstrated a good accuracy of 0.93%.

**Paper organization:** The paper is organized in a manner that thoroughly examines the study. The study begins with an introduction that provides an overview of the context and importance of the research. The following parts then provide a detailed explanation of the suggested approach. Subsequently, the study presents its findings, demonstrating the efficacy of the proposed methodology. Similarly, the study backs up its conclusions with visual aids and statistical measurements to help people understand better. The study will conduct a detailed assessment of its advantages and constraints and analyze its practical ramifications for future endeavors. Finally, the report provides a concise overview of the research's accomplishments and the study's prospective implications.

## 2 Related work

SS-DRPL has become a popular and interesting method in machine learning over the past few years (Ericsson et al., 2022). It may be better than traditional guided methods in some ways (Feng et al., 2019). However, when looking at how it can be used for more challenging tasks like predicting Parkinson's disease, the theories behind SS-DRPL seem less developed than those behind traditional guided learning paradigms (Zhang et al., 2017). Likewise, the goal of this review of the literature is to look into the pros and cons of using SS-DRPL to predict voice-based PD, emphasizing new research and evaluations that have been done recently. The basic idea behind normal supervised learning is to figure out how well a model should do on data it has yet to see (Jiang et al., 2020; Sen et al., 2020). On the other hand, SS-DRPL methods are hard because the training loss is usually optimized for a pretext task, but the downstream task's success metric is different. In addition, this misalignment brings up important questions about how learned models in SS-DRPL can be transferred and used in other situations. Because SS-DRPL has some problems when it comes to predicting Parkinson's disease, experts have done much real-world testing using different neural network architectures and datasets. For example, convolutional neural networks have been used in recent studies to sort large amounts of spectrogram images that contain gait patterns (Cetin, 2023; Nafiz et al., 2023). Deeply dense artificial neural networks have also been used to look at voice recordings and guess when Parkinson's disease will start or get worse.

The investigations conducted have yielded empirical findings that indicate encouraging results. The proposed models consistently surpass the current advanced methods in terms of categorization accuracy. Similarly, the VGFR spectrogram detector had a notable accuracy rate of 0.88, while the voice impairment classifier displayed an even better accuracy rate of 0.89 (Johri et al., 2019). This suggests the possibility of utilizing SS-DRPL approaches to improve predictive accuracy in the diagnosis and monitoring of PD. In addition, researchers have investigated different approaches to enhance the representation of features and the accuracy of classification in tasks related to predicting PD. Moreover, one way to do this is to use correlation maps made by principal component analysis, information gain, and other methods for feature selection to make the feature space bigger (Sharma and Mishra, 2022). It is important to note that the classification results obtained by using extended feature sets have been better than those obtained by using original features. This underscores the significance of feature engineering in augmenting prediction performance. More precisely, Researchers have investigated various supervised AI algorithms, such as Decision Trees, K-Nearest Neighbors, Random Forests, Bagging, AdaBoosting, and Gradient Boosting, in order to improve classifier accuracy and predictive performance in PD classification (Sabu et al., 2022; Shastry, 2023). These techniques utilize permutation computations to optimize model performance and improve the accuracy of receiver operating characteristic (ROC) curves, which is a crucial parameter for evaluating classifier performance.

In order to enhance the accuracy of feature representation and the results of classification, authors in Malekroodi et al. (2024) have implemented three separate component selection procedures. These strategies enable each of the 23 features to identify and pick the top 10 most effective features. The DT classifier has shown remarkable accuracy, reaching 0.94%, even in datasets with 23 features, similar to those with only 11 features. Specifically, the exceptional performance has been validated through the examination of the ROC curve, which has demonstrated a noteworthy area under the curve of 0.98. The implication of these findings are of great importance for clinical practice, as they highlight the effectiveness of computer-based algorithms in reliably differentiating people with PD from those without PD at the individual level. Clinicians can utilize advanced AI techniques and careful feature selection strategies to utilize computer-based findings. This enables them to improve patient care and outcomes in the management of PD by facilitating early diagnosis, personalized treatment planning, and ongoing disease monitoring. SS-DRPL opens up new ways to improve the prediction of voice-based PD. However, there are still some problems to solve before the theoretical foundations of SS-DRPL can be used in real-life medical tasks (Tripathi et al., 2024). Overall, by tackling these problems, SS-DRPL can be fully useful for better diagnosing, treating, and predicting Parkinson's disease, which will eventually improve patient outcomes and quality of life.

## 3 Proposed methodology

**Figure 1:**
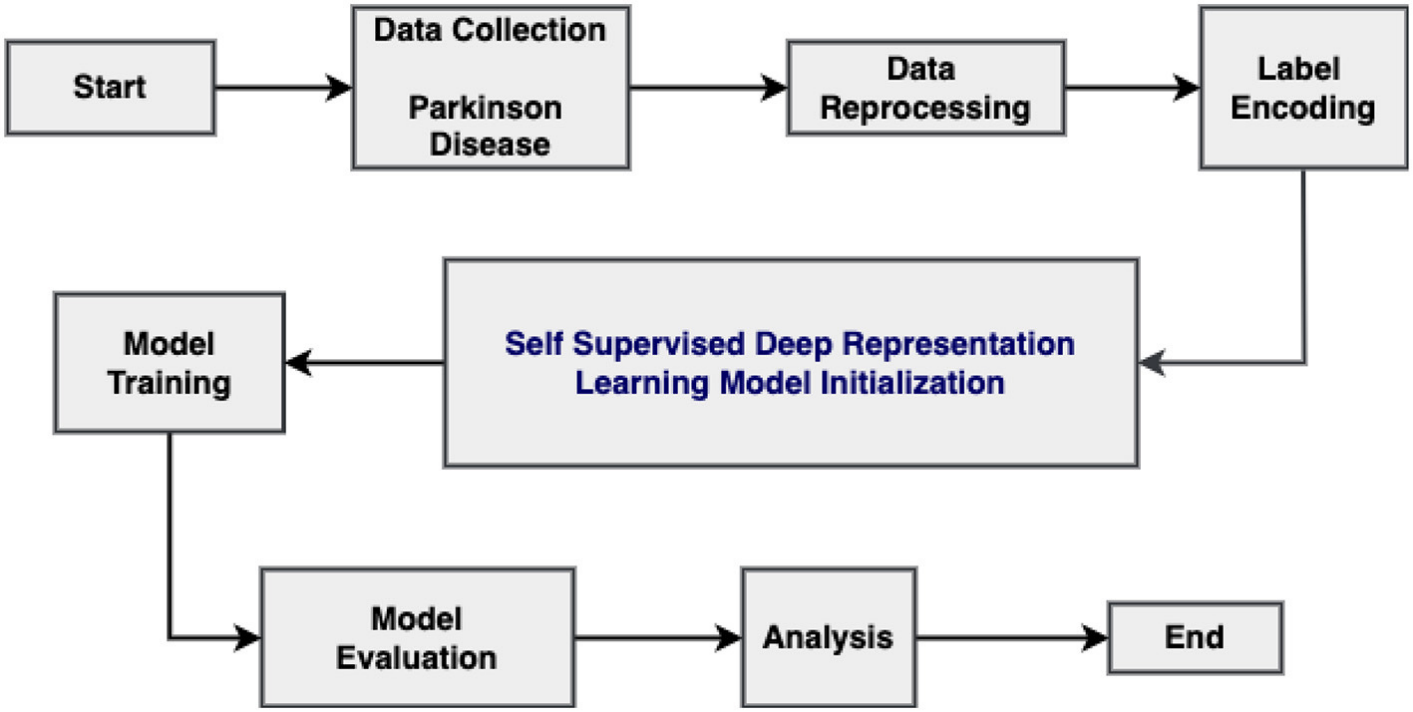
Employed methodology.

This section will provide an overview of the research design, paradigm, data collection methods, and analysis tools employed in the study. Ensuring accuracy in the approach is crucial for establishing the reliability and validity of the findings. Following that, this section provides a brief yet thorough summary of the strategic method, establishing the foundation for a thorough empirical investigation and significant contributions to the area. Figure 1 illustrates the methodology employed. The proposed approach starts with dataset collection, then pre-processing, then label encoding and the employing SSRL-based deep learning models for training and testing, and finally, results analysis.

The dataset used in this study consists of 707 voice samples obtained from individuals with various traumatic, biological, and neurological disorders, including Parkinson's disease, along with 53 samples from healthy individuals serving as controls. Each sample, averaging 2 s in duration, was recorded in controlled, quiet acoustic settings. The dataset encompasses multiple voice features such as fundamental frequency (MDVP:Fo), frequency variations (MDVP:Fhi, MDVP:Flo), jitter (MDVP:Jitter), shimmer (MDVP:Shimmer), noise-to-harmonic ratio (NHR), and harmonics-to-noise ratio (HNR), among others. These features provide comprehensive information about voice signals. Likewise, to ensure uniformity in feature scales, the data underwent preprocessing steps, including downsampling from 50 to 25 kHz and scaling using Min Max scaling (Ali et al., 2014). By bringing all features into a common numerical range, this preprocessing helps improve the convergence of machine learning models (Little et al., 2007). Algorithm 1 presents an overview of the entire pipeline of the proposed approach, which begins with collecting data, preprocessing it and evaluating results.

**Algorithm 1:**
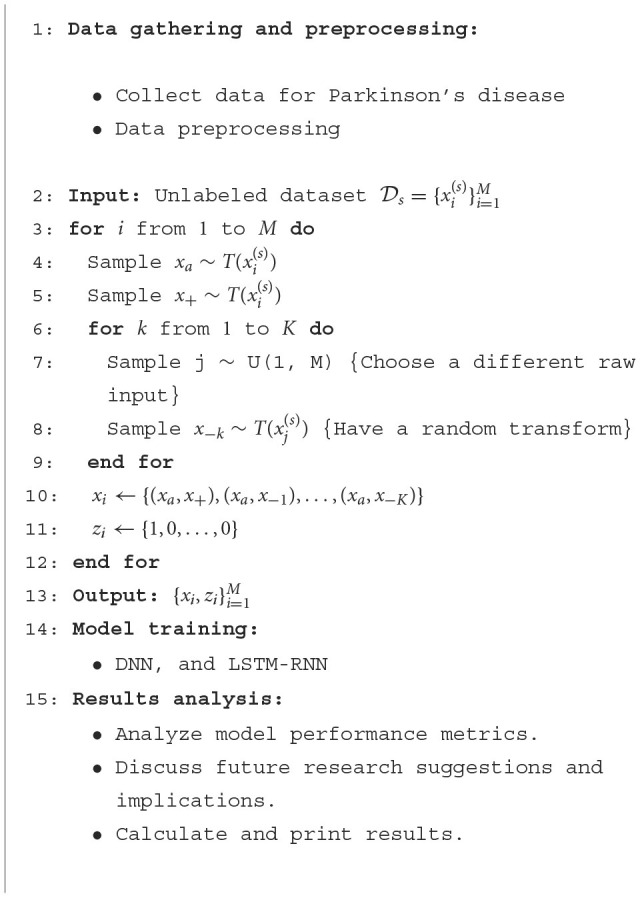
SS-DRPL for voice-based Parkinson's disease detection.

The utilization of conventional machine learning models, including Support Vector Machine (SVM), Decision Tree (DT), Logistic Regression (LR), and Gaussian Naive Bayes (GNB), did not incorporate SS-DRPL. Following this, the research employed SS-DRPL techniques, specifically DNN and LSTM-RNN. The classification of Parkinson's disease was performed by training these models using preprocessed voice samples, utilizing the retrieved features. The study utilizes a DNN structure consisting of numerous dense layers for representation learning and fully linked layers for self-supervised learning and transfer learning. To extract varied representations from input voice data, the model employs parallel, thick layers consisting of 256 neurons each. The representations are refined using separate, fully connected layers with 64 neurons and then flattened and concatenated for transfer learning. In order to do binary classification, a sigmoid activation function is employed in a final, fully linked layer consisting of 256 neurons. Subsequently, the model was made with the Adam optimizer and the binary cross-entropy loss function. Early stopping techniques were used to lower the risk of overfitting during training.

**Figure 2:**
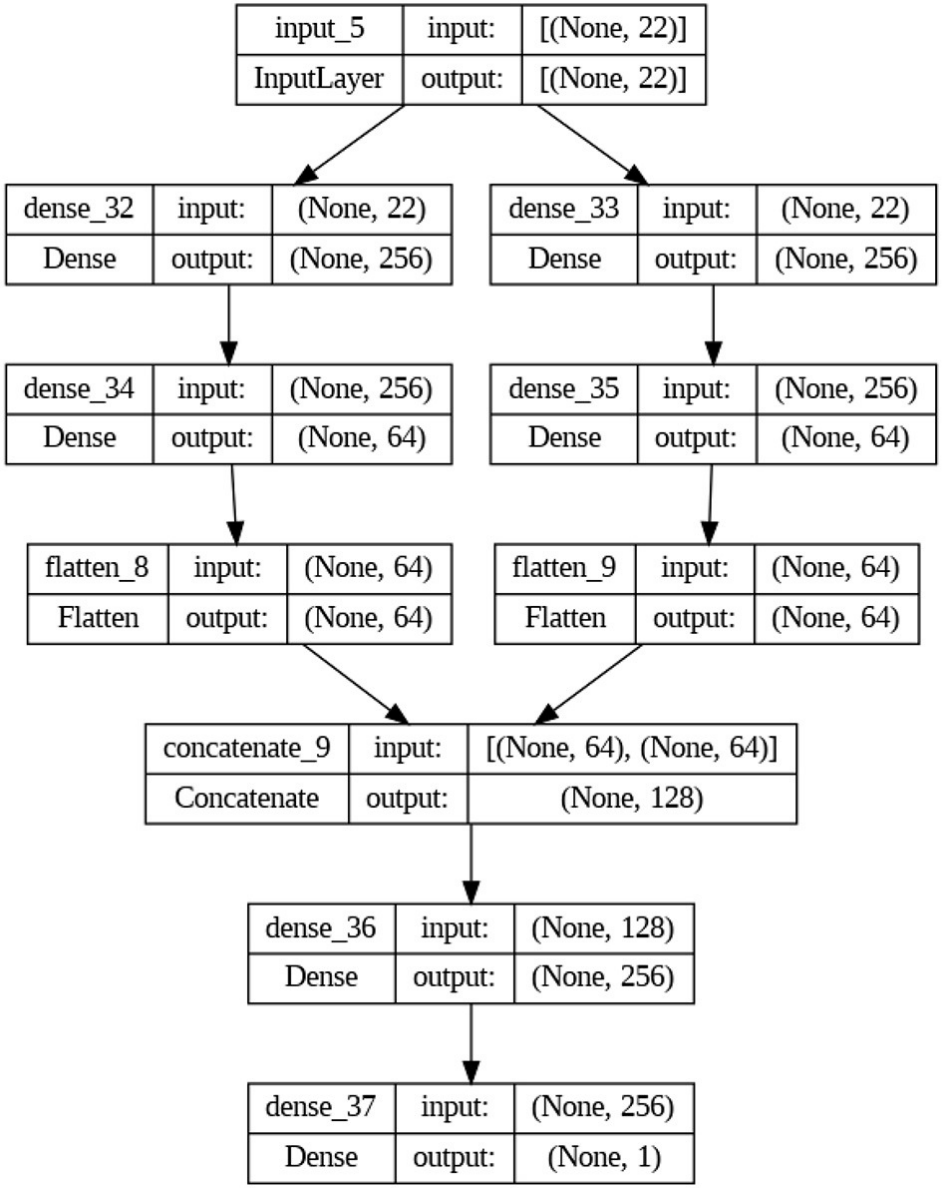
DNN architecture.

**Figure 3:**
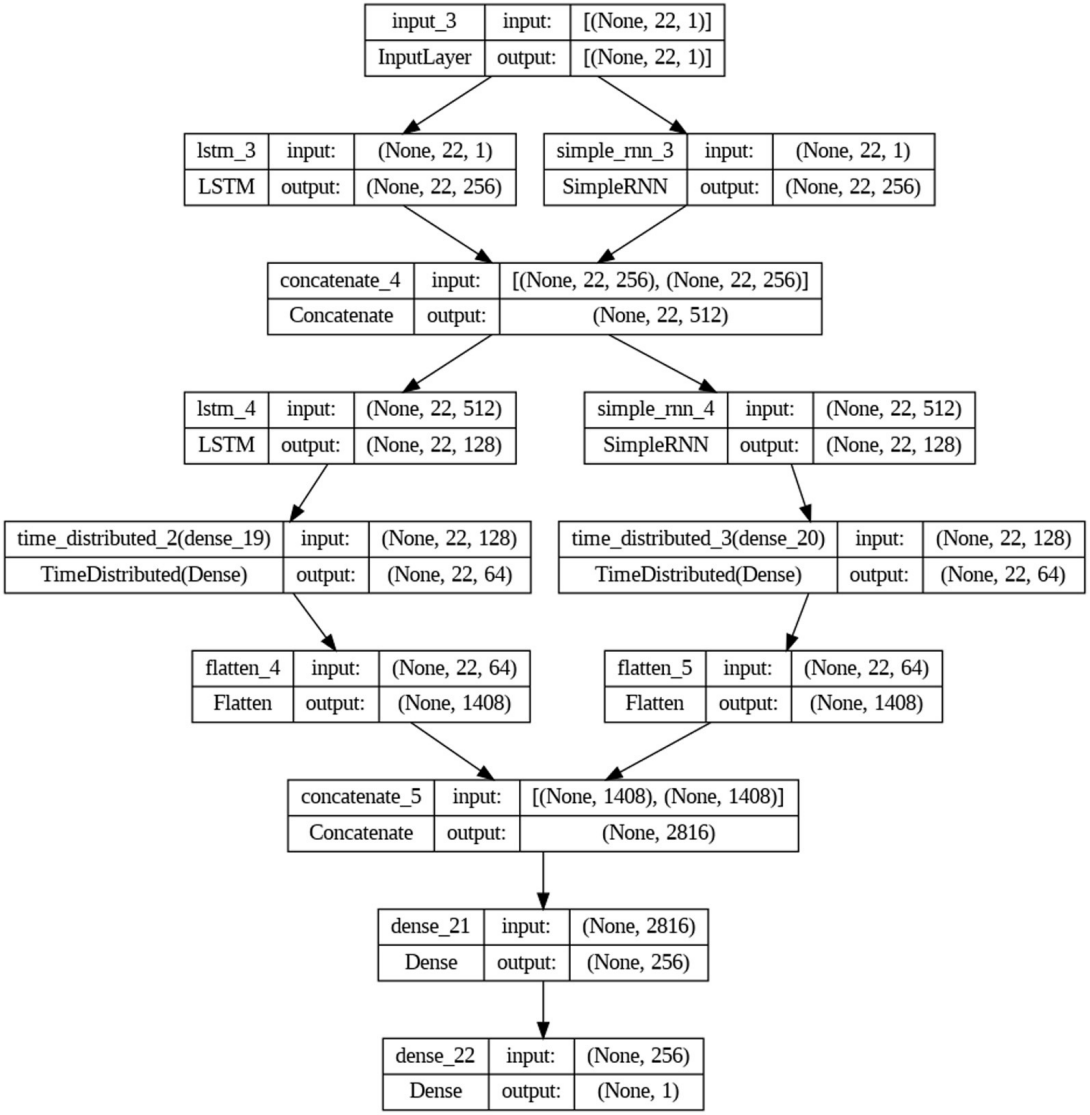
LSTM-RNN architecture.

The study also includes an LSTM-RNN architecture for learning representations. The suggested model has two layers, LSTM and RNN, running in parallel. Each layer has 256 neurons, and they work together to capture the temporal dependencies in the voice data. The outputs of the layers listed above are combined and then sent to the extra LSTM and RNN layers, each of which has 128 neurons, improving the accuracy of the representations. Self-supervised learning is implemented using fully connected layers consisting of 64 neurons each. These layers are applied using a time-distributed wrapper to process each time step separately. In the context of transfer learning, the outputs undergo a process of flattening and concatenation. Subsequently, fully connected layers with 256 neurons are employed for binary classification, utilizing a sigmoid activation function. The experimental setup involves training the model using the Adam optimizer and binary cross-entropy loss function. To mitigate the risk of overfitting, early halting is employed. Figures 2, 3 show the architectures of DNN and LSTM-RNN. These models possess the ability to independently acquire significant representations straight from unprocessed voice signals, obviating the necessity for manually designed features. In addition, the preprocessed samples were used to train the DNN and LSTM-RNN models. Throughout the training process, the models acquired the ability to extract complex characteristics from the unprocessed voice signals, effectively capturing detailed patterns that are known to be indicative of Parkinson's disease. The inherent characteristic of self-supervised learning allows the models to acquire representations independently without the need for explicitly labeled input to extract features.

Both classical machine learning models and self-supervised deep representation learning models were tested to see how well they worked. These tests included accuracy, precision, recall, F1-score, ROC curve, and confusion matrix analysis. This set of measures offers significant insights into the outcomes of the algorithms to detect Parkinson's disease based on speech samples precisely. SS-DRPL algorithms can assist in early diagnosis by improving the accuracy and reliability of detection methods. This enables healthcare practitioners to intervene during the early stages of the disease. By taking a proactive approach, not only are patient outcomes improved, but effective treatment techniques may be implemented promptly, eventually boosting the overall management of the condition.

## 4 Results and experimentation

The outcomes of the study are presented and interpreted in the results and experimentation section, taking into consideration the research objectives. Similarly, this part provides the results and offers valuable perspectives, explanations, and ramifications of the findings. The dataset was split into 80% for training and 20% for testing. Likewise, voice-based PD poses a complicated diagnostic dilemma owing to its diverse range of manifestations. The objective of this study is to utilize SS-DRPL in order to create reliable classification models that can effectively detect voice-based PD using a wide range of features derived from patient data. The study utilized several machine learning algorithms, such as DT, LR, SVM, and GNB, as well as deep learning algorithms, such as LSTM-RNN and DNN, each possessing distinct strengths and capabilities. The LSTM-RNN models exhibited outstanding performance, showcasing impressive precision, recall, and F1-score metrics, all reaching a value of 0.94. Using recurrent neural networks to find complex linkages in the data is a good way to classify Parkinson's disease cases accurately. The improved models demonstrate strong performance, which shows great potential for clinical applications. In these contexts, early and precise diagnosis plays a critical role in effectively managing diseases and implementing intervention measures. Table 1 shows the performance metrics of employed models.

**Table 1:** Classification reports for selected models.

**Model**	**Precision (%)**	**Recall (%)**	**F1-score (%)**	**Accuracy (%)**
LSTM-RNN	0.94	0.94	0.94	0.94
DNN	0.86	0.86	0.84	0.86
DT	0.85	0.86	0.85	0.86
LR	0.92	0.92	0.92	0.92
SVM	0.84	0.80	0.74	0.80
GNB	0.87	0.73	0.75	0.73

**Figure 4:**
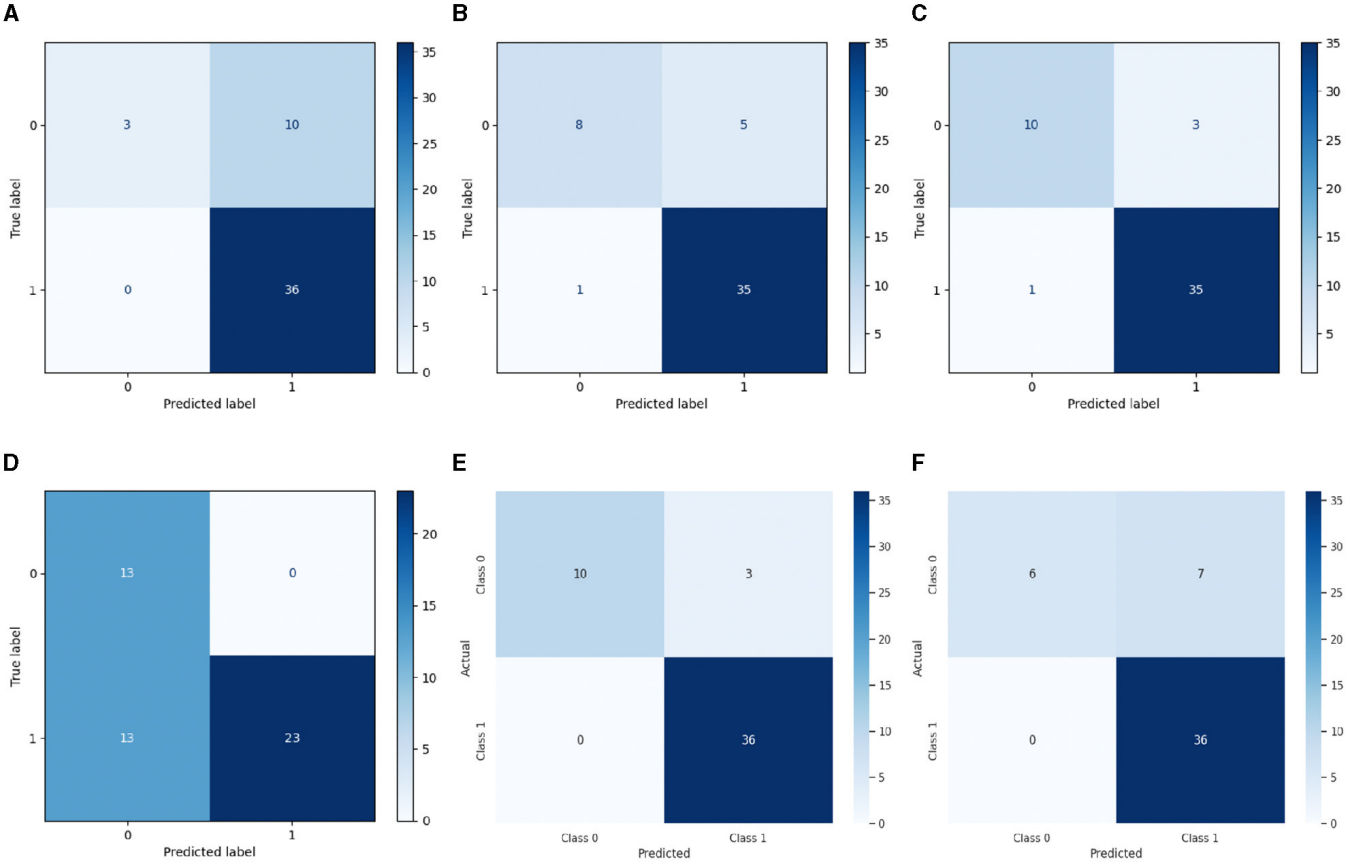
Confusion matrix of **(A)** SVM, **(B)** DT, **(C)** LR, **(D)** Gaussian NB, **(E)** LSTM-RNN, and **(F)** Deep DNN models.

**Figure 5:**
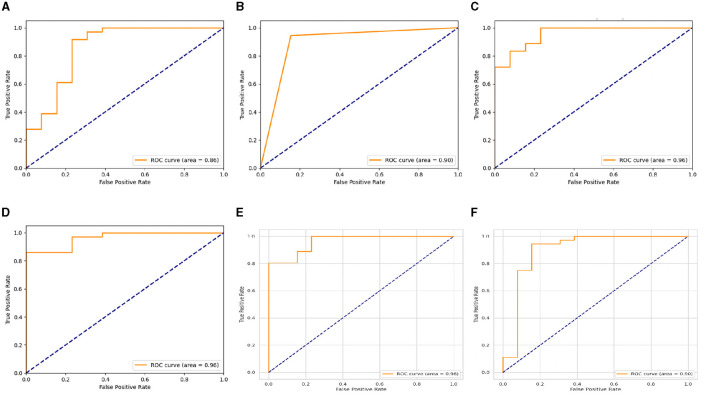
ROC curves of **(A)** SVM, **(B)** DT, **(C)** LR, **(D)** Gaussian NB, **(E)** LSTM-RNN, and **(F)** Deep DNN models.

Nevertheless, the LSTM-RNN demonstrated better performance; the DNN model displayed somewhat poorer precision, recall, and F1-score metrics, but it still achieved satisfactory levels of accuracy. Figures 4, 5 show the confusion matrix and ROC curves of the employed models. Subsequently, the observed disparity can be attributed to the model's proficiency in extracting pertinent features from the data or its aptitude for generalizing to unfamiliar examples. However, the performance of DNN highlights its potential usefulness in voice-based PD classification tasks, although there is still some opportunity for enhancement. Moreover, our examination of conventional machine learning algorithms showed various degrees of effectiveness. The Logistic Regression and Decision Tree models demonstrated high accuracy rates of 0.92 and 0.86, respectively, indicating their suitability for voice-based PD classification tasks. In contrast, the SVM and Gaussian NB models had somewhat lower percentages of accuracy, specifically 0.80 and 0.73, respectively. This indicates the necessity for further optimization or feature engineering in order to enhance their diagnostic capabilities for Parkinson's disease. These findings provide useful insights into the advantages and constraints of various machine learning methods for classifying Parkinson's disease.

**Figure 6:**
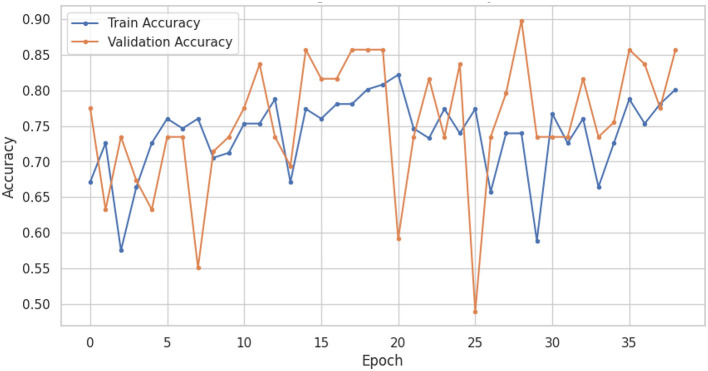
DNN training and validation accuracy curves.

**Figure 7:**
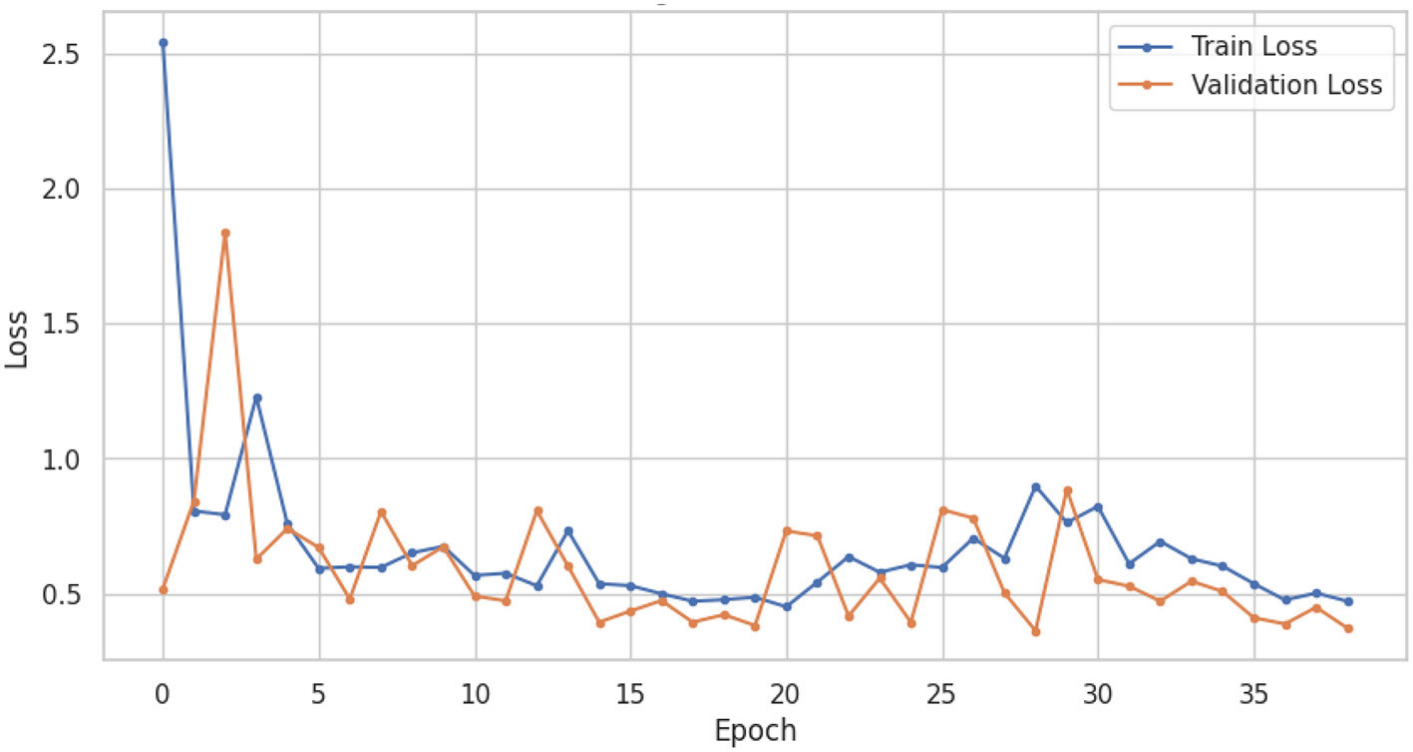
DNN training and validation loss.

The LSTM-RNN performs strongly, highlighting their potential usefulness in clinical environments where precise and prompt diagnosis is crucial for patient care. Also, comparing different types of traditional machine learning models gives us much useful information about how well they work and where we can find new research and development opportunities. In summary, this research showcases the considerable potential of machine learning in the categorization of voice-based PD, with sophisticated models like the LSTM-RNN exhibiting significant potential. Utilizing these models' advantageous aspects and acknowledging their constraints can provide a pathway toward enhanced voice-based PD, thereby leading to enhanced patient outcomes and improved quality of life. Figures 6, 7 shows the training and validation accuracy and loss curves provide a visual representation of the DNN performance during training, offering insights into its learning progress and potential overfitting or underfitting and assessing the convergence and generalization capabilities of the model, aiding in the optimization of hyperparameters and the identification of training issues.

**Figure 8:**
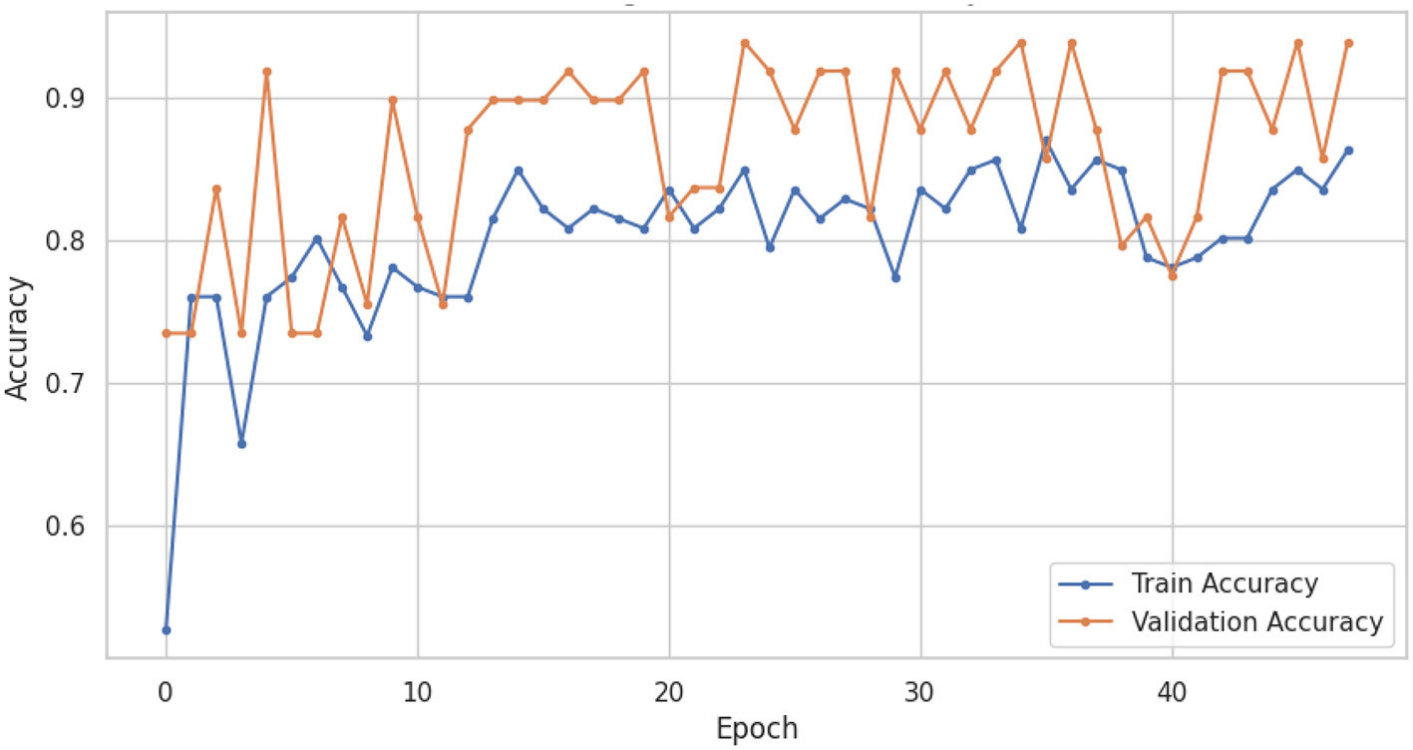
LSTM-RNN training and validation accuracy.

**Figure 9:**
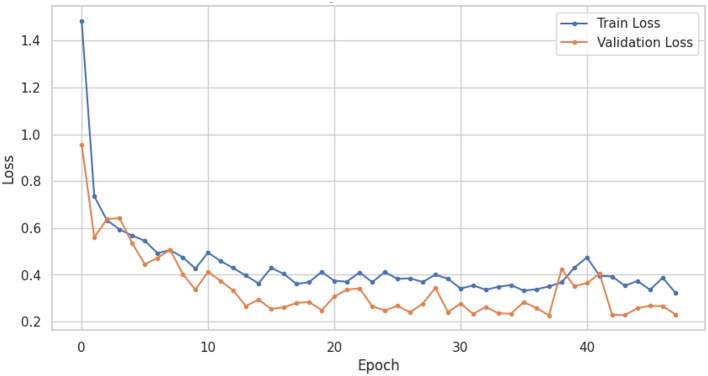
LSTM-RNN training and validation loss.

Figures 8, 9 show the training and validation accuracy and loss curves of LSTM-RNN performance during training, offering insights into its learning progress and potential overfitting or underfitting and assessing the convergence and generalization capabilities of the model, aiding in the optimization of hyperparameters and the identification of training issues.

Table 2 provides the comparison of the base study with our proposed approach SS-DRPL-based LSTM-RNN approach. The results reveal that the proposed approach provides the best accuracy of 0.94% in comparison with a base study suggesting our approach for voice-based PD detection.

**Table 2:** Comparison with base study.

**Study**	**Accuracy (%)**
LSTM-RNN	0.94
Arjmandi et al. (2011)	0.91

## 5 Conclusion and future work

This study explored the potential of deep learning techniques and SS-DRPL in voice-based PD classification. This study demonstrated the effectiveness of these models in identifying patterns within the dataset, thereby enabling accurate classification of PD individuals. Furthermore, the experiment of deep learning models yields significant findings regarding their relative effectiveness and possible applicability in clinical environments. Results reveal that the hybrid LSTM-RNN method effectively identifies voice-based PD using different sets of patient data features. Although this study offers useful insights into the classification of PD, there are still various areas for further research and development that need to be investigated. Similarly, combining different types of data sources, such as genetic markers, imaging data, and patient demographics, could improve the accuracy of classification models and give a fuller picture of the risk factors connected to PD. Furthermore, using longitudinal studies that track the progress of diseases over a long period could reveal important information about how voice-based PD develops and help the development of more personalized treatment methods. The establishment of collaborative partnerships among data scientists, physicians, and healthcare providers will play a crucial role in the translation of research findings into practical insights, thereby enhancing patient outcomes and advancing our comprehension of PD. This will ultimately result in a more precise diagnosis, tailored treatment strategies, and enhanced quality of life for individuals affected by this condition.

## Data availability statement

The original contributions presented in the study are included in the article/supplementary material, further inquiries can be directed to the corresponding authors.

## Author contributions

TK: Writing – original draft. MK: Writing – original draft. SO: Writing – original draft. GS: Writing – original draft. MA: Writing – original draft.
